# Carotid Artery Calcification Detected on Panoramic Radiography Is Significantly Related to Cerebrovascular Accident, Coronary Artery Disease, and Poor Oral Health: A Retrospective Cross-Sectional Study

**DOI:** 10.3390/dj12040099

**Published:** 2024-04-10

**Authors:** Anmol Brar, Katherine DeColibus, D. Shane Rasner, Angela R. Haynes, Frank Pancratz, Oreoluwa Oladiran, Semiu O. Gbadamosi, Adepitan A. Owosho

**Affiliations:** 1 Division of Oral Diagnosis, Department of Diagnostic Sciences, College of Dentistry, The University of Tennessee Health Sciences Center, Memphis, TN 38163, USA; 2 Department of Periodontology, College of Dentistry, The University of Tennessee Health Sciences Center, Memphis, TN 38163, USA; 3 College of Dentistry, The University of Tennessee Health Sciences Center, Memphis, TN 38163, USA; 4 College of Medicine, The University of Tennessee Health Sciences Center, Memphis, TN 38163, USA; 5 Department of Epidemiology, Robert Stempel College of Public Health & Social Work Florida International University, Miami, FL 33199, USA; 6 Department of Diagnostic Sciences, College of Dentistry/Department of Otolaryngology—Head & Neck Surgery, College of Medicine, The University of Tennessee Health Sciences Center, 875 Union Avenue, Memphis, TN 38163, USA

**Keywords:** stroke, transient ischemic attack, angina pectoris, myocardial infarction, dental radiographs, oral health, hypertension, hyperlipidemia, DMFT index

## Abstract

Panoramic radiography imaging modality is widely used by dentists for diagnosing dental and jaw conditions. It can also detect carotid artery calcification (CAC), indicative of calcified atherosclerotic plaques in the carotid arteries. This cross-sectional retrospective study at the University of Tennessee Health Science Center investigated the link between CAC identified on panoramic radiograph (PR) and cerebrovascular accident (CVA), coronary artery disease (CAD), and poor oral health. Data from 314 CAC patients collected from 2014 to 2023 included age at diagnosis, gender, and clinical histories of hypertension, hyperlipidemia, diabetes mellitus, CVA, CAD, and the decay, missing, and filled permanent teeth (DMFT) index. These patients were age- and gender-matched with non-CAC patients for analysis. The findings revealed high prevalences of hypertension (86.2%), hyperlipidemia (57.6%), diabetes mellitus (30.7%), CVA (15.5%), and CAD (28.7%) amongst CAC patients and the average DMFT index was 26.6. A comparative analysis of 276 matched controls demonstrated significant differences in hypertension (85.9% vs. 57.6%), hyperlipidemia (58.3% vs. 33.7%), diabetes (32.6% vs. 22.1%), CVA history (14.9% vs. 5.1%), CAD (26.1% vs. 9.8%), and DMFT scores (26.3 vs. 23.7), all indicating strong associations between CAC and these health conditions. The adjusted analysis showed that hypertension (aOR: 3.20 [95% CI: 2.06–5.07]), hyperlipidemia (aOR: 1.70 [95% CI: 1.14–2.50]), CVA (aOR: 2.20 [95% CI: 1.13–4.30]), and CAD (aOR: 2.10 [95% CI: 1.28–3.60]) were significantly associated with CAC. Notably, only 41.7% of the patients received a medical consultation after CAC detection on PR. It is crucial for dentists to refer patients for further evaluation.

## 1. Introduction

Panoramic radiography is a common imaging modality used for diagnosing jaw and teeth pathologies. Carotid artery calcification (CAC) can be detected on dental panoramic radiographs [1,2,3,4,5]. CAC detected on a panoramic radiograph (PR) was first described by Friedlander and Lande in 1981 [1]. The authors suggested that calcification in the carotid artery is a risk marker for stroke. CAC represents calcified atherosclerotic plaques in carotid vessels [1]. Atherosclerosis is a chronic inflammatory disease of the arterial vasculature characterized by a formation of plaques consisting of necrotic cores, accumulated modified fats, endothelial cells, leukocytes, smooth muscle cells, calcified regions, and foamy macrophages in the subendothelial intimal layer of large- and medium-sized arteries that eventually results in remarkable stenosis that restricts blood flow and causes tissue death [6]. Atherosclerosis is the underlying etiology for coronary artery disease (CAD) and cerebrovascular accident (CVA) [7]. CAD is the most common type of heart disease and the most common cause of mortality in the United States (U.S.) [8]. CVA is the leading cause of disability in the U.S. [8]. The risk factors for atherosclerosis that can be categorized as modifiable are hypertension, hyperlipidemia, diabetes mellitus, smoking, metabolic syndrome, and inflammation [7,9,10]. The non-modifiable risk factors are older age, male gender, familial history, and genetics [7,9,11,12].

CAC may present on PRs unilaterally or bilaterally. CAC may appear as one or more nodular, verticolinear, vessel-outlining, or irregular scattered opacities beneath and behind the angle of the mandible at the level of the C3 and C4 vertebrae. The gold standard reference imaging modalities for diagnosing CAC are Doppler/duplex ultrasonography and computed tomography angiography [13]. Other imaging modalities that can be used to identify CAC are cone beam computed tomography and cervical spine radiograph. The prevalence of CAC detection on PRs in the general population ranges from 2% to 31.57% [1,4,14,15,16,17,18,19]. Amongst patients 54 years and older, the prevalence reported was 21.68% [20], and amongst African American adult females, the prevalence reported was 24% [21]. The accuracy of PRs in the identification of CAC remains inconclusive [13,22,23]. A study of carotid endarterectomy with stenosis ≥ 75% in the internal carotid artery showed that 99% of the plaques were calcified and up to 84% of them were detected on PRs preoperatively [24]. While a positive correlation between CAC on PRs and carotid artery stenosis has been suggested [4,16], it is essential to note that calcifications do not necessarily indicate stenosis and not all plaque lesions are calcified.

A significant association between CAC detected on PRs and CVA, CAD, diabetes mellitus, hyperlipidemia, hypertension, smoking, older age, and male gender has been established in numerous studies, although it is noteworthy that in most cases, the relationship does not maintain statistical significance when assessed collectively [3,5,25,26,27,28]. Furthermore, multiple studies have indicated a significant correlation between CAC and periodontitis [29]. Bilateral vessel-outlining CAC detected on PRs has been shown to be significantly correlated with cardiovascular diseases [30,31]. However, research examining the link between CAC and the decayed, missing, and filled teeth (DMFT) index, as a measure of oral health status, remains limited. The DMFT index is the most significant indicator used to assess the oral health status [32]. Patients with a higher DMFT index are considered to have poorer oral health compared to patients with a lower DMFT index. Based on these reasons, the aim of this study was to evaluate if there is a relationship between patients with CACs detected on PRs and CVA, CAD, and poor oral health.

## 2. Methods

A ten-year retrospective cross-sectional study of adult patients diagnosed with CAC on PRs was performed by retrieving the records of all patients with the term “carotid artery calcification” or “carotid” or “calcification of the carotid artery” from the electronic health records of the College of Dentistry, University of Tennessee Health Science Center (COD-UTHSC), from 2014 to 2023. This study was approved by the UTHSC IRB # (23-09744-XM). CAC was defined as nodular or verticolinear masses, or irregularly scattered opacities beneath and behind the angle of the mandible at the level of C3 and C4 vertebrae. The inclusion criterion was the presence of CAC on PRs evaluated by the investigators. Exclusion criteria were absence of detectable CAC on PRs and the absence of PRs to review. To confirm the diagnoses, the PRs of all patients retrieved were evaluated by the authors (AB, KD, DSR, ARH, and AAO) independently, and then collectively. The following data were collected: age at diagnosis on radiograph, gender, known cardiovascular risk factors (history of hypertension, hyperlipidemia, diabetes mellitus), CVA [stroke and transient ischemic attack], CAD [angina pectoris and myocardial infarction], and oral health status. The oral health status of patients was evaluated using the decayed, missing, and filled teeth (DMFT) index. A tooth may be missing for the following reasons: periodontal loss, decay, or trauma. The presence of an implant was counted as a missing tooth, and a tooth that was crowned was also counted as a filled tooth.

### 2.1. Control Group

A control group of patients without CAC randomly matched for exact age and gender (non-modifiable risk factors for atherosclerosis) with patients with CAC (cases) was retrieved from the electronic health records of COD-UTHSC for analysis. The matching of these patients was performed by an independent investigator (FP), who was blinded to the cases’ data sets, to avoid bias. To confirm the absence of CAC, the PRs of all control group patients were evaluated by the authors (KD, DSR, and AAO) and a similar data set was collected: age at diagnosis on radiograph, gender, history of hypertension, hyperlipidemia, diabetes mellitus, CVA, CAD, and DMFT index.

### 2.2. Statistical Analysis

All analyses were conducted using SPSS version 29.0 (IBM Corp., Armonk, NY, USA). First, we used descriptive statistics to characterize patients with CAC detected on PRs. Second, we compared the unilateral and bilateral presence of CAC by demographics, cardiovascular risk factors, cerebrovascular accident, coronary artery disease, and DMFT index using a two-sided *t*-test, chi-squared, or Fisher’s exact test when appropriate. Third, we compared the presence and absence of CAC by demographics, cardiovascular risk factors, cerebrovascular accident, coronary artery disease, and DMFT index using a two-sided *t*-test, chi-squared, or Fisher’s exact test when appropriate. Finally, using multivariable logistic regression, we estimated adjusted odds ratios (aORs) with 95% confidence intervals (CIs) for CVA and CAD associated with CAC. The following variables were considered confounders such as age and gender by matching, and hypertension, hyperlipidemia, and diabetes mellitus were included in the regression analysis. A statistical significance is considered at a *p*-value < 0.05.

## 3. Results

**Figure 1:**
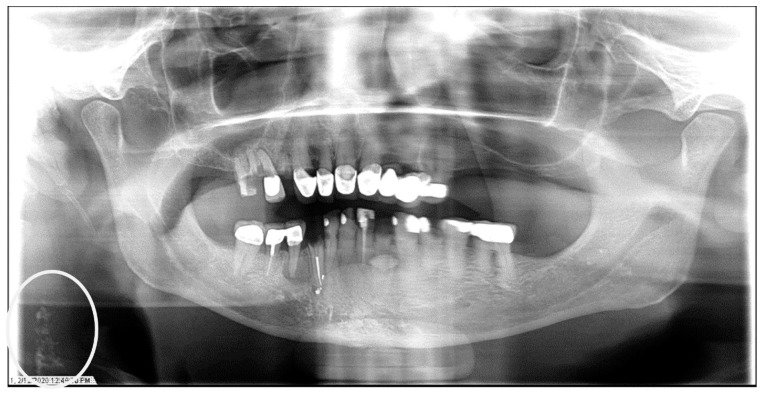
The panoramic radiograph of a 67-year-old male patient with a medical history of hypertension, diabetes mellitus, and coronary artery disease showing a unilateral right carotid artery calcification (CAC) during a comprehensive dental examination. The CAC is encircled by a white line.

**Figure 2:**
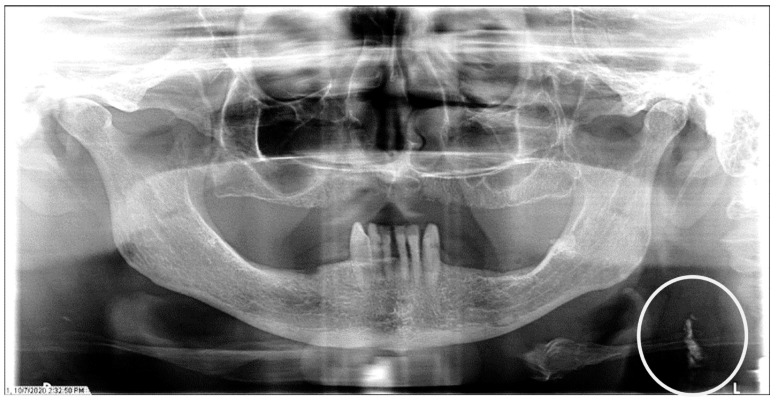
The panoramic radiograph of a 77-year-old female patient with a medical history of hypertension, hyperlipidemia, diabetes mellitus, and coronary artery disease showing a unilateral left carotid artery calcification (CAC) during a comprehensive dental examination. The CAC is encircled by a white line.

**Figure 3:**
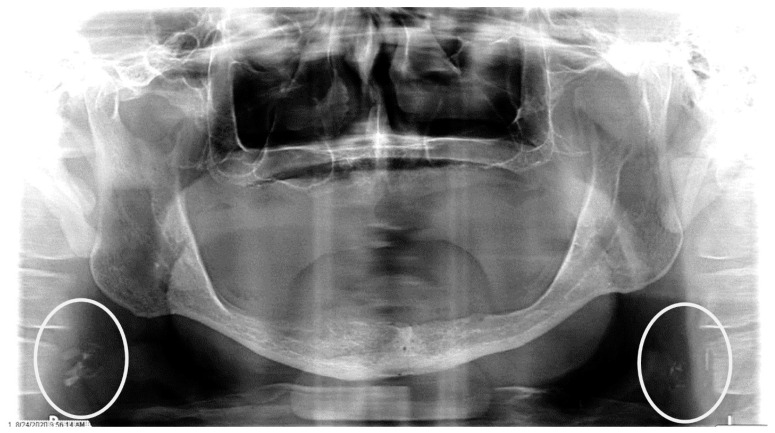
The panoramic radiograph of an 80-year-old male patient with a medical history of hyperlipidemia and diabetes mellitus showing bilateral carotid artery calcifications (CACs) during a comprehensive dental examination. The CACs are encircled by a white line.

**Figure 4:**
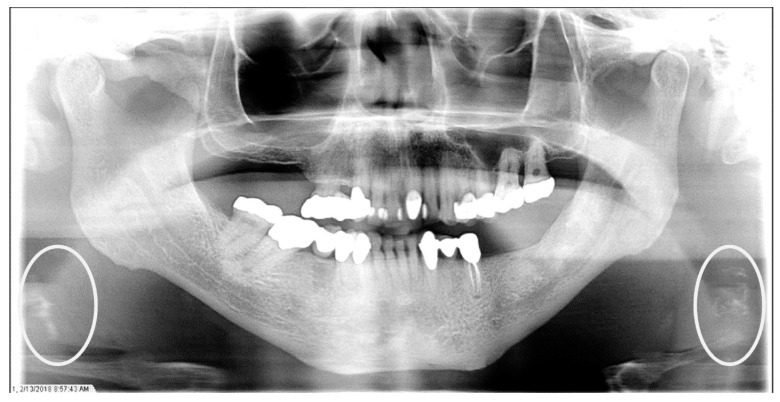
The panoramic radiograph of a 73-year-old male patient with a medical history of hypertension, hyperlipidemia, diabetes mellitus, and cerebrovascular accident showing bilateral carotid artery calcifications (CACs) during a comprehensive dental examination. The CACs are encircled by a white line.

**Figure 5:**
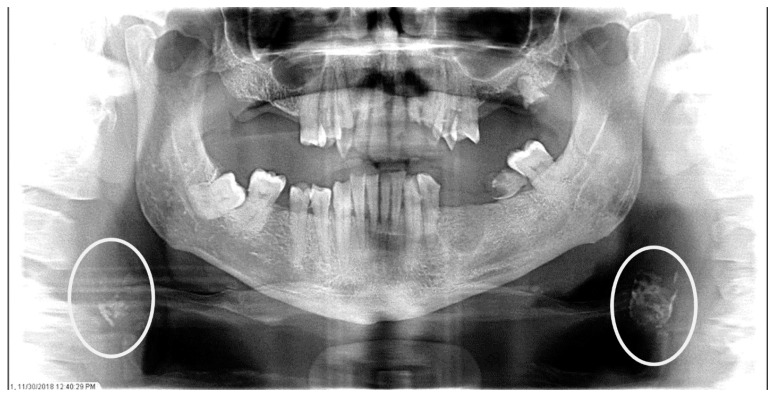
The panoramic radiograph of a 53-year-old male patient with a medical history of hypertension and hyperlipidemia showing bilateral carotid artery calcifications (CACs) during a comprehensive dental examination. The CACs are encircled by a white line.

The characteristics of the CAC patients are presented in Table 1. Between 2014 and 2023, there were 559 patient records with the terms “carotid artery calcification” or “carotid” or “calcification of the carotid artery” noted. After the panoramic radiographs were reviewed, 314 patients were confirmed to have a diagnosis of CAC (including 26 patients with a history of carotid endarterectomy). The remaining 245 patient records were excluded because of the absence of detectable CAC on PRs and the absence of PRs to review. The ages ranged from 29 to 92 years, with a median of 68 years. CAC was most prevalent (39.5%) in the seventh decade of life. There were 168 (53.5%) female and 146 (46.5%) male patients. The median age of the female patients was 68 years, and the median age of the male patients was 69.5 years. The calcifications were identified unilaterally in 168 (53.5%) patients (88 females/80 males; mean age: 67.8 years) (Figure 1 and Figure 2) and bilaterally in 146 (46.5%) patients (80 females/66 males; mean age: 68.6 years) (Figure 3, Figure 4 and Figure 5). The DMFT index in the CAC patients ranged from 8 to 32 (mean = 26.6). The frequency of patients with CAC with a history of hypertension, hyperlipidemia, diabetes mellitus, CVA, and CAD was 86.2%, 57.6%, 30.7%, 15.5%, and 28.7%, respectively.

There was no statistically significant difference between patients with unilateral and bilateral CAC detected on PRs when comparing age (*p* = 0.465), gender (*p* = 0.734), cardiovascular risk factors [hypertension (*p* = 0.622), hyperlipidemia (*p* = 1.00), and diabetes mellitus (*p* = 0.387)], CVA (*p* = 0.116), CAD (*p* = 0.802), and DMFT index (*p* = 0.234) (Table 2). Two hundred seventy-six CAC patients (150 females and 126 males; mean ± [SD] age of 68.5 ± [8.7] years) were matched by exact age and gender with 276 control patients. Of the 276 CAC patients, 237 were hypertensive, 161 had hyperlipidemia, 90 were diabetic, 41 had a history of CVA, and 72 had a history of CAD. In comparison, of the 276 control patients, 159 were hypertensive, 93 had hyperlipidemia, 61 were diabetic, 14 had a history of CVA, and 27 had a history of CAD (Table 3). Descriptive analyses revealed that the CAC patients were significantly associated with hypertension (*p* < 0.001), hyperlipidemia (*p* < 0.001), diabetes mellitus (*p* = 0.007), CVA (*p* < 0.001), CAD (*p* < 0.001), and the DMFT index (*p* < 0.001) (Table 3). A multivariable logistic regression analysis adjusted for covariates revealed that hypertension (aOR: 3.2, [95% CI: 2.06–5.07], *p* < 0.001), hyperlipidemia (aOR: 1.7, [95% CI: 1.14–2.50], *p* < 0.001), CVA (aOR: 2.2, [95% CI: 1.13–4.30], *p* < 0.001), and CAD (aOR: 2.1, [95% CI: 1.28–3.60], *p* < 0.001) were significantly associated with the presence of CAC on PRs (Table 4). One hundred thirty-one patients of the 314 (41.7%) CAC patients had records for medical consultation or referral to their physician on account of the CAC detected on PRs.

## 4. Discussion

Every year in the U.S., around 1.6 million patients (about the population of West Virginia) are diagnosed with either CAD or CVA. Approximately 87% of the patients diagnosed with stroke are diagnosed with the ischemic type, secondary to blockage from atherosclerotic plaque. About 20% of all heart attacks are silent. In 2021, over 375,000 people died from CAD in the U.S. The monetary impact of heart disease and stroke on the U.S. economy between 2018 and 2019 was about $296.5 billion (*This information was assessed on the CDC website, 25 January 2024*). The early identification of at-risk individuals may help reduce the burden of these diseases. Atherosclerosis is a systemic condition that mostly affects the aortic, coronary, carotid, and leg vessels. The detection of atherosclerosis in one region of the body may be a telltale sign of the conditions of large- and medium-sized arteries in other areas of the body. PRs, which are routinely used in dental clinics all over world, have been shown to detect CAC [1]. CAC is a calcified atherosclerotic plaque in the carotids, most commonly at the bifurcation, where the common carotid artery divides into internal and external carotid arteries. Although not 100% accurate, mostly due to false negatives [13], the identification of an incidental CAC on a PR during a routine dental visit should initiate a referral to a physician for further evaluation, as supported by the American Dental Association Council on Advocacy for Access and Prevention. The Council also advocates carotid ultrasound scans to be performed by dental hygienists. These ultrasound scans take little chair time in a dental office. This shows that dental providers have a role in the interprofessional collaborative management of patients.

Our study is the first to show a significant correlation between CAC detected on PRs and the DMFT index, as a representation of oral health status. The patients with CAC exhibited a significantly higher DMFT index. Multiple studies have shown a significant correlation between CAC detected on PRs and periodontitis [29]. The association between CAC and the DMFT index may heavily rely on the number of missing teeth. Periodontitis, if untreated, results in tooth loss. The study by Desvarieux et al. showed that tooth loss was significantly related to the prevalence of carotid plaques and the study by Schillinger et al. showed that tooth loss was not only a significant predictor for the prevalence of carotid stenosis but also for a worsening progression of the disease [33,34]. Inflammation is a well-known modifiable risk factor for atherosclerosis and inflammation from chronic periodontitis has been suggested as a possible etiology for atherosclerosis. This interdisciplinary perspective underscores the importance of holistic healthcare, recognizing the intricate connections between oral hygiene and systemic well-being.

This study also establishes a significant relationship between CAC detected on PRs and hypertension, hyperlipidemia, diabetes mellitus, cerebrovascular accident, and coronary artery disease. Even after addressing confounders on a multivariable logistic regression analysis adjusting for covariates, cerebrovascular accident, and coronary artery disease were found to be significantly related to CAC detected on PRs. The relative risk of CAC formation is increased by 220% in a hypertensive patient, 70% in a patient with hyperlipidemia, 120% in a patient with CVA, and 110% in a patient with CAD. The reverse may also be true in that the identification of CAC on PRs significantly increases the likelihood of hypertension, hyperlipidemia, cerebrovascular accident, or coronary artery disease in a patient.

The significant correlation between CAC detected on PRs and hypertension have been reported in other studies. The study by Mӧst et al. reported the relative risk of CAC detection on dental radiographs is increased by 66.6% in hypertensive patients [3]. The study by Abecasis et al. reported that hypertensive patients were 5.426 times more likely to have CAC detected on PRs [35]. Also, studies by Kumagai et al., Basuga et al., Johansson et al., and Moshfeghi et al. have shown significant independent correlation between CAC detected on PRs and hypertension [5,25,26,36]. Hyperlipidemia is a well-known risk factor for atherosclerosis and has also been shown to be significantly related to CAC detected on PRs [3,7,9,25]. The study by Mӧst et al. also showed that the relative risk of CAC detection on dental radiographs is increased by 64.9% in patients with hyperlipidemia; in patients with both hypertension and hyperlipidemia, the relative risk of CAC detection on dental radiographs is increased by 122% [3]. The study by Kumagai et al., also reported a significant correlation between CAC detected on PRs and hyperlipidemia even after addressing confounders (age, gender, hypertension, diabetes mellitus) on a multivariate logistic regression analysis [25]. Our study found a significant independent correlation between CAC detected on PRs and diabetes mellitus on a univariate analysis, as reported in other studies by Mӧst et al., Kumagai et al., Aghazadehsanai et al., and Johansson et al. [3,21,25,36].

Stroke and myocardial infarction (MI) are among the most severe complications of atherosclerosis. It has been suggested that atherosclerosis affects both carotid and coronary arteries synchronously [37,38]. Several studies have discussed the relationship between CAC detected on PRs and CVA [15,30,39,40,41]. The study by Kwon et al. showed that the prevalence of CAC detected on PRs in stroke patients is significantly higher than in non-stroke patients [2]. The significant correlation between CAC detected on PRs and MI has been reported in other studies [26,36,42]. The study by Johansson et al. showed that a history of MI was significantly prevalent in patients with CAC detected on PRs compared to non-CAC patients [36]. Also, the study by Gustafsson et al. reported the relative risk of CAC detection on PRs increased by 24% in MI patients compared to patients without MI and was more conspicuous in patients with bilateral CAC involvement [42]. The study concluded that CAC detection on PRs could serve as a risk marker for future MIs [42]. However, a systematic review evaluating the role of PRs in the detection of CAC as a predictor for CAD or CVA concluded that it was unclear but may aid the identification of at-risk patients who need further medical assessment [43]. In the review, most patients with CAC detected on PRs were overall more likely to develop a future CAD or CVA event compared to a control group; nonetheless, the majority of the studies did not show a statistical significance [43].

The study by Carasso et al. showed that the detection of CAC on PRs was correlated with high coronary artery calcium and tripled the probability of having a clinically significant coronary artery calcium score that warrants clinical evaluation [44]. Coronary artery calcium scoring is a widely available, specific, and readily reproducible means of assessing risk for coronary artery disease and cardiovascular events; a great assessment tool for asymptomatic individuals for planning preventive strategies. The study by Donders et al. showed a significant independent correlation between an elevated coronary artery calcium score and tooth loss on a univariate analysis but was not significant on a multivariable analysis [45]. These studies further show the significance of the identification of CAC on PRs and how it relates to CAD and its correlation to oral health. Dental providers should be more apt in the detection of CAC on PRs and, if identified or suspected, should have patients evaluated further. The limitations of this study are its retrospective cross-sectional nature. The precise timeline for the various events cannot be evaluated and the appropriate sequence of events cannot be evaluated. PRs are not the gold standard for the detection of CAC and the misinterpretation of other calcified structures in that region, such as calcified cervical lymph nodes and calcified thyroid cartilage, as CAC is possible. We did not validate the use of dental panoramic radiographs for detecting CAC by comparing with another imaging modality due to the retrospective cross-sectional nature of this study. Also, the calculation of DMFT was solely based on the radiographic assessment and review of clinical charts. Information such as the precise reason for tooth loss could not be ascertained. Larger prospective multi-institutional longitudinal studies are recommended to ascertain the sequential correlation of CAC detected on PRs and CVA, CAD, and their risk factors. Further correlations between CAC detected on PRs and the coronary artery calcium score should be investigated.

## 5. Conclusions

We analyzed the clinical characteristics of 314 patients with CAC detected on PRs. We performed an analysis on 276 test cases matched by non-modifiable risk factors of atherosclerosis (age and gender) of a control group and found that CAC detected on PRs was significantly related to CVA, CAD, and their associated modifiable risk factors: hypertension, hyperlipidemia (on a multivariable analysis), and diabetes mellitus (on a univariate analysis). Also, this is the first study to report a significant relationship between CAC detected on PRs and a higher DMFT index (poor oral health status) compared to those without CAC. The incidental finding of CACs on PRs is evidence of disease progression. Therefore, dentists are highly encouraged to make appropriate referrals of patients with CAC to be evaluated for cerebrovascular accident, coronary artery diseases, and their risk factors.

## Figures and Tables

**Table 1:** Clinical characteristics of patients with carotid artery calcifications detected on panoramic radiograph (N = 314).

Characteristics	N (%)
Sex	
Female	168 (53.5)
Male	146 (46.5)
Age (years)	
Range	29–92
Median	68
Age distribution	
0–29	1 (0.3)
30–39	2 (0.6)
40–49	3 (1)
50–59	40 (12.7)
60–69	124 (39.5)
70–79	109 (34.7)
80–89	32 (10.2)
90–99	3 (1)
Location	
One side	168 (53.5)
Both sides	146 (46.5)
DMFT	
Range	8–32
Average	26.6
Hypertension	
Yes	268 (86.2)
No	43 (13.8)
Hyperlipidemia	
Yes	178 (57.6)
No	131 (42.4)
Diabetes mellitus	
Yes	95 (30.7)
No	214 (69.3)
Cerebrovascular accident	
Yes	48 (15.5)
No	261 (84.5)
Coronary artery disease	
Yes	89 (28.7)
No	221 (71.3)

**Table 2:** Comparisons between patients with unilateral and bilateral carotid artery calcification(s) by age, gender, cardiovascular risk factors, cerebrovascular accident, coronary artery disease, and DMFT index.

	Unilateral	Bilateral	*p*-Value
Age (mean ± SD) years	67.8 ± 9.1	68.6 ± 9.2	0.465 *
Gender (F; M)			0.734
Female	88	80	
Male	80	66	
Hypertension			0.622
Yes	142	126	
No	25	18	
Hyperlipidemia			1.00
Yes	96	82	
No	70	61	
Diabetes mellitus			0.387
Yes	55	40	
No	111	103	
Cerebrovascular accident			0.116
Yes	31	17	
No	135	126	
Coronary artery disease			0.802
Yes	49	40	
No	117	104	
DMFT index (mean ± SD)	26.96 ± 5.7	26.17 ± 6	0.234 *

*—*p*-value calculated by two-sided *t*-test, the remaining *p*-values were calculated by chi-squared test.

**Table 3:** Comparisons between patients with and without carotid artery calcification by cardiovascular risk factors, cerebrovascular accident, coronary artery disease, and DMFT index after matching.

	CAC N = 276	Control N = 276	*p*-Value
Age (mean ± SD; range) years	68.5 ± 8.7; (34–92)	68.5 ± 8.7; (34–92)	1.00 *
Gender (F; M)			1.00
Female	150	150	
Male	126	126	
Hypertension			<0.001
Yes	237	159	
No	39	117	
Hyperlipidemia			<0.001
Yes	161	93	
No	115	183	
Diabetes mellitus			0.007
Yes	90	61	
No	186	215	
Cerebrovascular accident			<0.001
Yes	41	14	
No	235	262	
Coronary artery disease			<0.001
Yes	72	27	
No	204	249	
DMFT index (mean; range)	26.3; (8–32)	23.7; (2–32)	<0.001 *

*—*p*-value calculated by two-sided *t*-test, the remaining *p*-values were calculated by chi-squared test.

**Table 4:** Multivariable logistic regression estimating adjusted odds ratios (aORs) with 95% confidence intervals (Cis) for factors associated with CAC (N = 552).

	aOR	95% CI	*p*-Value
Hypertension	3.20	2.06–5.07	<0.001
Hyperlipidemia	1.70	1.14–2.50	0.009
Diabetes mellitus	0.85	0.55–1.30	0.465
CVA	2.20	1.13–4.30	0.02
CAD	2.10	1.28–3.60	0.004

## Data Availability

The data are unavailable due to privacy or ethical restrictions. Please contact the corresponding author.
